# Meaning in Life: A Major Predictive Factor for Loneliness Comparable to Health Status and Social Connectedness

**DOI:** 10.3389/fpsyg.2021.627547

**Published:** 2021-02-24

**Authors:** Dídac Macià, Gabriele Cattaneo, Javier Solana, José M. Tormos, Alvaro Pascual-Leone, David Bartrés-Faz

**Affiliations:** ^1^Departament de Medicina, Facultat de Medicina i Ciències de la Salut i Institut de Neurociències, Universitat de Barcelona, Barcelona, Spain; ^2^Oficina de Recerca i Innovació, Institut Guttmann, Institut Universitari de Neurorehabilitació adscrit a la UAB, Badalona, Spain; ^3^Departament de Biomedicina, Facultat de Medicina i Ciències de la Salut i Institut de Neurociències, Universitat de Barcelona, Barcelona, Spain; ^4^Departament de Medicina, Universitat Autónoma de Barcelona, Bellaterra, Spain; ^5^Hinda and Arthur Marcus Institute for Aging Research and Deanna and Sidney Wolk Center for Memory Health, Hebrew SeniorLife, Boston, MA, United States; ^6^Department of Neurology, Harvard Medical School, Boston, MA, United States

**Keywords:** loneliness, meaning in life, sense of coherence, mental health, social isolation

## Abstract

**Objective:** Loneliness is the subjective distress of feeling alone and has a strong impact on wellbeing and health. In addition to well-known predictors like isolation and poor health, a better understanding of the psychological determinants of loneliness would offer effective targets for future complementary interventions.

**Methods:** In this cross-sectional observational study (*N* = 2,240), we compared the explanatory power of several important risk factors of loneliness with the affective, motivational, and cognitive aspects of the Meaning in Life (MiL) construct. Different nested linear models were compared including socio-demographic, lifestyles, social-connectedness, and self-rated health variables, to assess the overlapping and non-overlapping explanatory power of each of them.

**Results:** Health status and MiL were found to be the most important predictors of loneliness, followed by social connectedness and, with a much lower weight, lifestyles, and socio-demographic factors. Within the MiL factor, the most cognitive component, sense of coherence, had a greater explanatory power than the more affective and motivational ones.

**Conclusion:** Reduced MiL, the capacity of an individual to attach “value and significance” to life, is a crucial predictor to the feeling of loneliness. These results suggest that programs aiming to combat loneliness should go well beyond situational interventions and include more cognitive, value-centered interventions that enable individuals to define and pursue a meaningful vital plan.

## Introduction

Loneliness is often defined as the feeling that one's desired quantity or quality of social connections is unfulfilled (Peplau and Perlman, 1982; Hawkley and Cacioppo, 2010). Loneliness is universal and may have evolved in our species as a signaling mechanism to change behavior and avoid dangerous isolation (Cacioppo and Patrick, 2008). However, loneliness is also strongly subjective, involving a judgement of the meaning and adequacy of one's social connections that necessarily presupposes an individual's cognitive framework of values and expectations. This is why similar social configurations (e.g., being single, having few friends, working alone, etc.) in different people, cultures, or even historical periods often give rise to very different feelings of loneliness (Peplau and Perlman, 1982; Rokach et al., 2001; Klinenberg, 2016; Snell, 2017). Thus, loneliness is distinct from “social connectedness” which refers to a more objective situational condition involving number, diversity, and frequency of connections (De Jong Gierveld, 1998). In fact, distinguishing between loneliness and social connectedness also makes sense from a health perspective, since the causal pathways linking them to infirmity or higher mortality are partially independent (Rokach et al., 2001; Cacioppo et al., 2009; Shankar et al., 2011; Boss et al., 2015).

Over the past decades, the prevalence of loneliness has sky-rocketed (Snell, 2017) probably as the consequence of accelerated profound changes in family structure, workplace relationships, digital connectedness, sedentarism, and urban social lifestyles, all of which affect not only the quantity and quality of social interactions but also our values and expectations about them. Recent surveys report that between one and two out of every ten adults often or always feel lonely in the US, Japan, and Europe (D'Hombres et al., 2018; Dijulio et al., 2018).

There is no longer a debate about the public health relevance of high and persistent loneliness. Lonely people are more likely to present health problems, including higher rates of depression and anxiety, cognitive impairment, and dementia, as well as poorer physical health, including motor dysfunctions, hypertension, cardio- and cerebro-vascular diseases, obesity, and sleep problems (Cacioppo et al., 2002; Tabue Teguo et al., 2016; Sutin et al., 2018). Loneliness also increases the risk of unhealthy lifestyles like sedentarism, smoking, or substance abuse, which in turn aggravate its deleterious load on health (Dyal and Valente, 2015; Vancampfort et al., 2019).

The growing interest of public health actors to combat loneliness has mainly focused on social connectedness, health status (especially mental health), and socio-economic variables to define risk groups and intervention strategies. However, less attention has been paid to potential cognitive and psychological determinants whilst the International Loneliness and social Isolation research NetworK (I-LINK) has recently emphasized “[loneliness] interventions must be tailored and matched to specific root causes of loneliness” (Fried et al., 2020).

However, studying the psychological determinants that, given an individual's social configuration, leads to loneliness has a long history. Researchers have pointed to numerous predisposing personality traits like low self-esteem, shyness, introversion, self-consciousness, resilience, or optimism (Perlman and Peplau, 1973). Recently, more cognitively based constructs have also been associated to loneliness: wisdom including personal and cultural values such as higher spirituality, compassion, or mastery (Ben-Zur, 2018; Morlett Paredes et al., 2019).

Here, we aimed to assess the contribution of meaning in life (MiL), differentiating between its cognitive, affective, and motivational subcomponents, to predicting loneliness and compared it to other well-known determinants (sociodemographic, lifestyle, health, and social connectedness). The choice is motivated because it is the lack of “meaning” in social interactions, more than number or frequency, that is often pinpointed as the key determinant of loneliness.

MiL is defined as the capacity of a person to attach value and significance to his or her life (Steger, 2012). It was originally defined and assessed as a single construct (Steger et al., 2006), however, more recent studies have shown the idoneity of a finer-grained partition into three subcomponents (Martela and Steger, 2016), which can be differently associated to relevant health outcomes (Bartrés-Faz et al., 2018): (1) A cognitive aspect, coherence, capturing the ability of ‘understanding one's life and the external world and how one fits in it’, that we measured with the Sense of Coherence scale (SoC; Antonovsky, 1993). Antonovsky defined SoC as a “global orientation that expresses the extent to which one has a pervasive, enduring confidence of (a) *comprehensibility*, i.e., that the stimuli deriving from one's internal and external environments in the course of life are structured, predictable, and explicable; *manageability* (b) i.e., the resources are available to one to meet the demands posed by these stimuli; and *meaningfulness* (c) i.e., these demands are challenges, worthy of investment and engagement.” Epidemiological and clinical investigations have provided convincing evidence that higher SoC is associated with greater stress coping capacity and that it represents an important health-promoting resource, both regarding positive health perception (Eriksson and Lindström, 2006) and quality of life across all age ranges (Huang et al., 2017). A strong SoC has been shown to predict subjective well-being to a greater extent than physical disabilities in older individuals (Schneider et al., 2006) and be associated with reduced mortality, lower incidence of depression, and better cognitive function (Read et al., 2005). Specifically, in the case of loneliness, for example, SoC was found to mediate the negative effects of loneliness on feelings of hope amongst bereaved parents (Einav and Margalit, 2020).

(2) A motivational aspect, purpose, related to “long-term life goals, and aspirations that motivate behavior,” that we measured with the Purpose in life subscale (PiL; Ryff, 1995). PiL is conceptualized as having a purposeful sense of direction, and related to “long-term life goals and aspirations that motivate behavior.” PiL has been linked to a reduced risk of health threatening conditions such as stroke (Kim et al., 2013), cardiovascular events, as well as with all-cause mortality (Cohen et al., 2016). PiL has also been associated to greater engagement in positive lifestyles such physical activity (Hooker and Masters, 2016) and reduced sleep problems (Kim et al., 2015). In older age, PiL is strongly related with mental and physical health, and individuals with medium and high PiL levels exhibit lower health care utilization and expenditures, showing higher social support and resilience, and higher quality of life (Musich et al., 2018). Finally, PiL has been associated with better cognitive function in adults without dementia, and with reduced risk of dementia or mild cognitive impairment (Boyle et al., 2010).

(3) Finally, a more affective aspect, significance, linked to the idea of engagement, satisfaction, and fulfillment, that we measured with the Engagement with life scale (EwL; Trompetter et al., 2013). This MiL component relates to an individual's evaluation of how valuable, worthwhile, and important life is. Previous evidences showed that this dimension is associated with increased mental health by also promoting well-being and reducing psychological distress (Ho et al., 2010), the risk of depressive symptoms (Mascaro and Rosen, 2005), and the impact of depression in post-traumatic stress disorder patients (Owens et al., 2009). This affective component is frequently targeted by psychological therapies (Zhang et al., 2018) in order to identify meaningful values, promote behavioral changes in line with it, promote mental health, and reduce depression (Trompetter et al., 2013).

Our interest in studying the contribution of MiL in feelings of loneliness lies in the fact that MiL components are relatively modifiable and amenable to interventions and education in values. In particular, cognitive-behavioral therapy-based approaches can focus on the identification of life values and the reinforced orientation of behavior toward them (Zhang et al., 2018). Furthermore, MiL components (in particular SoC and PiL) have been related to a myriad of physical and mental conditions, including the ones frequently reported amongst lonely people such as anxiety, depression, cognitive or functional impairment with advanced age, and also risk for cerebro- or cardio-vascular events (Eriksson and Lindström, 2006). Therefore, insights into the role of the affective, motivational, and cognitive dimensions of MiL could suggest novel interventions to combat loneliness.

## Methods

### Participants

Study subjects were volunteer participants of the Barcelona Brain Health Initiative (BBHI, www.bbhi.cat/en), a prospective longitudinal cohort initiated in Barcelona (Spain) in 2017 and aimed to identify and characterize the lifestyle factors, biological determinants, and their interactions, related to maintenance of mental and brain function across the lifespan (Cattaneo et al., 2018). Our study includes a subsample of 2,240 (mean age 54.3, standard deviation 7.3, range 40–68, 66.1% women) of the total BBHI registered participants (*n* = 5,100, mean age 53.4, standard deviation 7.0, range 40–68, 66.7% women, using all registered participants as of February 2019). Inclusion criteria obey the following rules: (1) Participants had completed all of the necessary questionnaires regarding sociodemographic, lifestyle, social connectedness, and health related variables (detailed below) through the dedicated BBHI web-based platform (2,388 were selected). Socio-demographic, lifestyles, self-rated health, social connectedness, and loneliness questionnaires were collected in the one year follow-up questionnaire. As for MiL subcomponents, we used specific questionnaires administered at the end of the first wave (for more details, see Cattaneo et al., 2020). A number of these questionnaires (e.g., loneliness, MiL, and certain lifestyles) were not collected repeatedly in the two waves, thus preventing a longitudinal approach. (2) All respondents were free from neurological or psychiatric medical diagnoses (148 participants had to be excluded from the 2,388 with completed questionnaires). The study was approved by the *Comité d'Ètica i Investigació Cl*í*nica de la Unió Catalana d'Hospitals* and all participants gave their informed consent.

### Data Collection Instruments

#### Loneliness

The main outcome variable of our study was estimated using the Spanish version of the widely employed three-item UCLA scale, adapted for large surveys (Hughes et al., 2004; Rico-Uribe et al., 2016). It consists of three items in which participants have to rate the frequency of several experiences (“How often do you feel isolated from others?,” “How often do you feel excluded?,” and “How often do you feel that you lack company”) on a 3-point Likert scale (options: “1: Hardly ever,” “2: Some of the time,” or “3: Often”). By summing up the score of each of the three items, we obtained a quantitative scale ranging from 3 to 9. The psychometric properties of the 3-Item UCLA Scale in our sample (mean = 3.76, sd = 1.20, alpha = 0.77) were comparable to the ones obtained by Hughes et al. (2004) from a similar sample employed to validate their reduced scale. Following these authors, we maintained the continuous quantitative nature of the additive final score treating it as an interval magnitude.

#### Predictive Factors

We investigated potential predictive variables of loneliness grouped into five conceptually distinct groups: (1) socio-demographic, (2) lifestyles, (3) social connectedness, (4) general health, and (5) meaning in life. Sample characteristics are provided in Table 1. Extensive references to the literature for every questionnaire and scale can be found in the published BBHI protocol (Cattaneo et al., 2018).

**Table 1:** Potential risk factors for loneliness included in the study with sample characteristics.

**Predictors**	**Sample characteristics**
**1. Socio-demographic**	
**1.1. Sex**	Female (66.1%), Male (33.9%)
**1.2. Age**	54.26 (sd = 7.15)
**1.3. Level of Education**	Primary (3.5%), Secondary (23.1%), Higher ed. (73.4%)
**1.4.Family income corrected by family size and structure** (times above the corresponding poverty threshold)	2.61 (sd = 1.15)
**2. Lifestyles**	
**2.1. Nutrition**. Mediterranean Diet Adherence screener (MeDAS)	8.20 (sd = 1.94)
**2.2. Exercise**. Physical Activity Questionnaire (IPAQ)	Low act. (18.8%), Moderate act. (42.4%), High act. (38.8%)
**2.3. Cognitive Activity**. Classical proxies of cognitive stimulating activities	13.56 (sd = 3.72)
**2.4. Sleep Quality**. Jenkins Sleep Evaluation Questionnaire (JSEQ)	8.02 (sd = 3.61)
**3. Social connectedness**	
**3.1. Household Arrangement**	Alone (15.8%), With partner (70.9%), Living without partner but with children in charge (8.3%), Other (5.0%)
**3.2. Social Interaction**. 4 question from the LUBBEN Social Network Scale (LSNS).	13.94 (sd = 3.54)
**4. Self-rated general health**	
**4.1. Cognitive Health**. From Neuro-QoL questionnaire, including 12 questions on memory, attention, and reasoning.	51.23 (sd = 8.04)
**4.2. Mental Health**. From the PHQ-4 questionnaire, consisting of 2 questions on depressive, 2 on anxiety symptoms.	14.1 (sd = 1.93)
**4.3. Physical Health**. 3 questions from the General Health PROMIS questionnaire, of which 2 relate to physical disability and 1 to physical well-being in general.	8.49 (sd = 1.28)
**5. Meaning in life**	
**5.1. Sense of Coherence**. Abbreviated version of the Orientation to Life Questionnaire (OLQ-13)	66.24 (sd = 11.22)
**5.2. Purpose in Life**. Six-item subscale of the Spanish version of Ryff's Well-Being Scale	28.95 (sd = 5.7)
**5.3. Engagement with Life**. Trompetter's scale (2013) to assess fulfillment and personal values	61.61 (sd = 9.18)

##### Sociodemographic

(1) sex, (2) age, (3) level of education (primary, secondary, and higher), and (4) family income corrected by family size and structure by normalizing the raw family income by the corresponding poverty threshold given size and family structure according to the Statistical Institute of Catalonia (IDESCAT).

##### Lifestyles

(1) Nutrition was assessed using the Spanish version of the Mediterranean Diet Adherence Scale (Schröder et al., 2011), a 14-item (scoring 0 or 1) instrument that assess baseline adherence to Mediterranean diet, where 12 questions ask about food consumption frequency (vegetables, tomato, rice, pasta, nuts, sugar-sweetened beverages, etc.) and two questions about food consumption habits (olive oil and chicken as principal fat and meet source). (2) Exercise was measured using the Spanish version of the International Physical Activity Questionnaire (IPAQ; Román Viñas et al., 2013), a self-reported scale of physical activity by duration and frequency encompassing several domains (job-related, housework, leisure-time, and others). Results are reported in three main levels of physical activity: Low (inactive adults), Moderate, and High. (3) Cognitive Activity was estimated using seven items from the short Cognitive Reserve Scale (CRS) (Rami et al., 2011) involving the training/information and hobbies spheres of the Cognitive Reserve. These items directly ask about the degree of involvement in learning foreign languages, playing or listening to music, reading, writing, watching TV, painting, and other cognitively stimulating activities. (4) Sleep: The Spanish adaptation of the Jenkins Sleep Evaluation Questionnaire (Jenkins et al., 1988; Pallarés-Sanmartín et al., 2019) was used to assess sleep quality in the last month. The four items asking about sleep problems in falling asleep or staying asleep, awakenings during the night and waking up tired and worn out.

##### Social Connectedness

(1) Household Arrangement is a four-level factor specifying with whom a person lives (alone, with partner with or without children in charge, without partner but with children in charge or other). (2) Social Interaction includes four out of the 12 items from the Spanish version of the LUBBEN Social Network Scale (Lubben, 1988; Vilar-Compte et al., 2018), two of which involve relatives, the other two involve friends: “How many relatives/friends are you with or do you hear from, at least, once a month?,” “How often do you meet or hear from the relative/friend you have the most contact with?.” The remaining eight questions were excluded because they contain clearly subjective appreciations of social isolation that conceptually overlap with our outcome variable.

##### Self-Rated General Health

(1) Mental Health was measured with the ultra-brief self-reported Patient Health Questionnaire (PHQ-4), consisting of two questions on depressive and two on anxiety symptoms (Kroenke et al., 2009). The PHQ-4 Spanish version has been found to have good psychometric properties and to be sensitive to treatment-related changes in a validation study (Mills et al., 2015). (2) Cognitive Health was measured with the Spanish version of the Neuro-QoL questionnaire (Correia et al., 2015). This is a 12-item questionnaire for self-perceived memory, attention, and reasoning using a 5-point Likert scale. (3) Physical Health was measured using three questions from the Spanish version of the General Health PROMIS questionnaire (Ader, 2007; Hahn et al., 2014), of which two relate to physical disability and one to physical well-being in general.

##### Meaning in Life

(1) Sense of Coherence was measured using the abbreviated version of the Orientation to Life Questionnaire (OLQ-13). This 13-item scale represents a short version of the 29-item original scale proposed by Antonovsky (1993) and has been previously validated in the Spanish population (Virues-Ortega et al., 2017). (2) Purpose in Life was measured using the subscale of the Spanish short version of the Ryff's Well-Being Scale (Díaz et al., 2006). (3) Finally, Engagement with Life was assessed with the Spanish translation of the 16 item- Trompetter's scale (2013).

## Data Analysis

We modeled the explanatory contribution of each risk factor to loneliness in a series of successive regression models using ordinary least squares. This allowed us to clarify their adjusted (conditional on other risk factors being fixed) and unadjusted contributions. In particular, each potential risk factor was associated with loneliness according to the following set of conditionings: (a) without controlling for any other predictive factor (this tends to be the option adopted in many public health and economic surveys), (b) controlling for risk factors within the same conceptual group, (c) controlling for risk factors belonging to other conceptual groups, and (d) controlling for all risk factors, both from within and without the same conceptual group. It is expected that the proportion of variance explained by each predictor as we increasingly adjust for other risk factors will decrease (unless masking effects appear) owing to the high collinearity between some of them.

**Figure 1:**
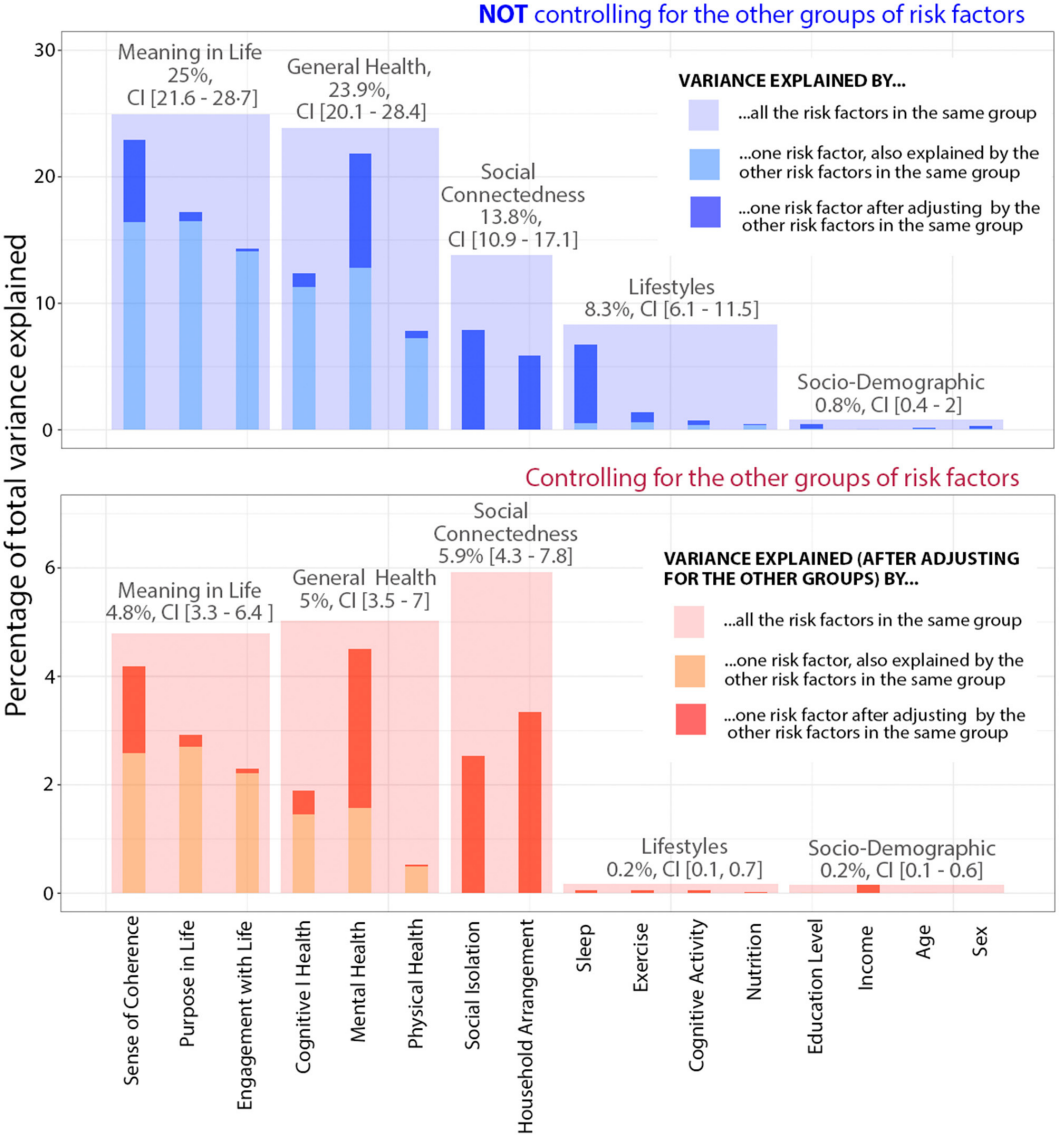
Proportion of variability in loneliness explained by different risk factors in models with different number of controlling factors. The full model including all risk factors classified in five conceptually distinct groups (including 16 factors) was able to explain a total of 37.87% of the variability in loneliness levels [adjusted R^2^ = 37.31%, CI (34.40%, 41.75%)].

**Figure 2:**
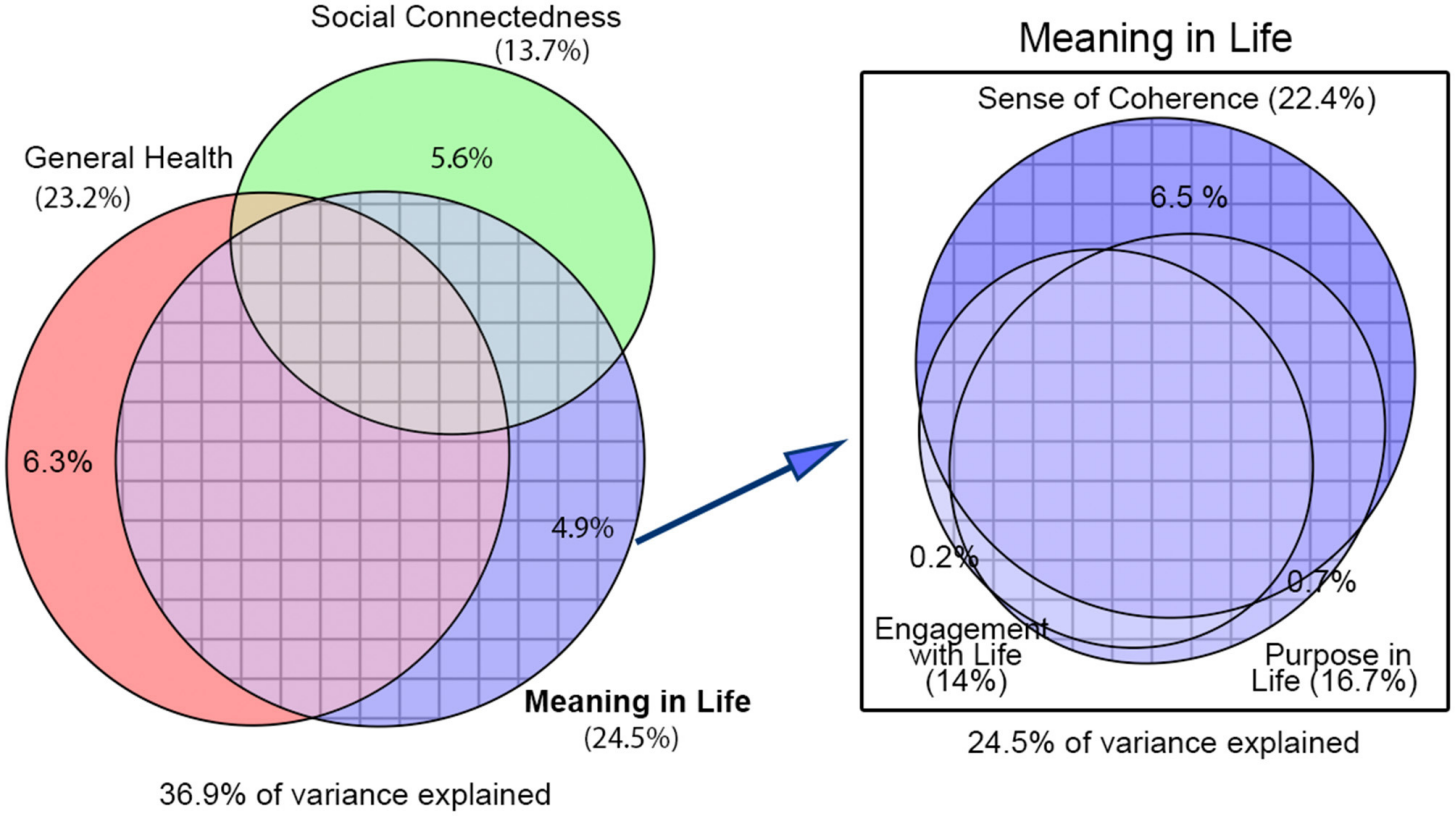
Elliptical Venn diagrams with overlapping and non-overlapping areas proportional to the total variance in loneliness explained by each group of risk factors. Figures in parentheses correspond to the explained variance when we do not control for the other risk factors represented (total area of the ellipses). Figures without parentheses correspond to the variance when we control for them (non-overlapping areas of the ellipses). Socio-demographic factors have always been partialed out to account for sampling selection biases in our study.

The explanatory contribution (goodness of fit) of each predictor or group of predictors was assessed with the adjusted coefficient of full (R^2^) or partial determination (Rp^2^), depending on whether other controlling covariates were included in the model (see Table 2). We used the adjusted version of R^2^ and Rp^2^ to penalize for the loss of degrees of freedom and make models comparable to one another. By contrast, in our visual representations (bar plots in Figure 1 and Venn diagrams in Figure 2), we did not plot adjusted R^2^ or Rp^2^, but directly plotted percentages of variance to make sure that overlapping and non-overlapping areas added correctly. However, note that the relationship between the proportion of total variance explained by a set of regressors and their corresponding adjusted R^2^ is very close but not identical. The departure of the partial Rp^2^ can be still greater because this statistic is defined as the ratio of the variance explained over the variance left unexplained by the adjusting regressors and not over the total variance.

**Table 2:** Explanatory contribution for each risk factor in regression models with increasing number of adjusting factors.

**Adjusted by risk factors in other groups**		**NO**				**YES**			
**Adjusted by risk factors in the same group**		**NO**		**YES**		**NO**		**YES**	
	***Df***	***R^**2**^ (%)***	**CI**	***Rp^**2**^ (%)***	**CI**	***Rp^**2**^ (%)***	**CI**	***Rp^**2**^ (%)***	**CI**
**Socio-demographic**	**5**	**0.60**	[0.15, 1.72]^*^	**-**	**-**	**0.04**	**[-0.11, 0.88]**	**-**	**-**
Age	1	0.27	[−0.02, 0.91]	0.19	[−0.04, 0.77]	−0.04	[−0.04, 0.17]	−0.04	[−0.04, 0.2]
Sex	1	0.13	[−0.04, 0.61]	0.06	[−0.04, 0.53]	−0.04	[−0.04, 0.17]	−0.04	[−0.04, 0.19]
Education	2	−0.08	[−0.09, 0.28]	−0.08	[−0.09, 0.26]	0.16	[−0.07, 0.78]	0.16	[−0.05, 0.77]
Income	1	0.41	[0.02, 1.2]^*^	0.30	[−0.02, 0.99]	−0.04	[−0.04, 0.23]	−0.04	[−0.04, 0.19]
**Lifestyles**	**5**	**8.09**	[5.88, 11.09]^*^	**–**	**–**	**0.07**	**[−0.1, 0.96]**	**–**	**–**
Nutrition	1	0.44	[0.02, 1.19]^*^	0.04	[−0.04, 0.45]	−0.01	[−0.04, 0.31]	−0.02	[−0.04, 0.28]
Cog. activity	1	0.71	[0.16, 1.58]^*^	0.35	[0, 0.98]	0.04	[−0.04, 0.46]	0.02	[−0.04, 0.42]
Exercise	2	1.31	[0.52, 2.58]^*^	0.80	[0.21, 1.87]^*^	0.00	[−0.08, 0.53]	0.01	[−0.08, 0.56]
Sleep	1	6.68	[4.46, 9.15]^*^	6.29	[4.19, 9.04]^*^	0.05	[−0.04, 0.51]	0.04	[−0.04, 0.53]
**Social connectedness**	**4**	**13.65**	[10.97, 17]^*^	**–**	**–**	**8.54**	[6.39, 11.4]^*^	**–**	**–**
Household structure	3	5.74	[3.89, 7.98]^*^	6.25	[4.16, 8.7]^*^	4.79	[3.01, 7.13]^*^	5.05	[3.29, 7.5]^*^
Social Interaction	1	7.89	[5.52, 10.38]^*^	8.39	[6.06, 10.9]^*^	3.68	[2.02, 5.4]^*^	3.94	[2.33, 5.87]^*^
**General health**	**3**	**23.80**	[19.9, 28.06]^*^	**–**	**–**	**7.37**	[5.07, 10.43]^*^	**–**	**–**
Physical health	1	7.79	[5.62, 10.28]^*^	0.71	[0.13, 1.82]^*^	0.75	[0.11, 1.86]^*^	0.01	[−0.04, 0.45]
Mental health	1	21.78	[18, 25.87]^*^	10.55	[7.59, 14.2]^*^	6.67	[4.4, 9.29]^*^	4.46	[2.63, 6.82]^*^
Cognitive health	1	12.34	[9.29, 15.76]^*^	1.34	[0.46, 2.71]^*^	2.78	[1.32, 4.68]^*^	0.67	[0.06, 1.81]^*^
**Meaning in life**	**3**	**24.87**	[21.62, 28.45]^*^	**–**	**–**	**7.04**	[4.87, 9.67]^*^	**–**	**–**
Engagement with Life	1	14.30	[11.42, 17.63]^*^	0.24	[−0.04, 0.85]	3.38	[1.9, 5.19]^*^	0.07	[−0.04, 0.52]
Purpose in life	1	17.21	[13.81, 20.58]^*^	0.94	[0.22, 2.06]^*^	4.32	[2.53, 6.49]^*^	0.31	[−0.03, 1.09]
Sense of coherence	1	22.90	[19.52, 26.52]^*^	7.93	[5.65, 10.43]^*^	6.21	[4.15, 8.54]^*^	2.48	[1.28, 4.22]^*^

*All coefficients of determination are adjusted to take into account the loss of degrees of freedom associated to each risk factor and thus penalize overfit. Whenever other controlling risk factors are included (Models NO-YES, YES-NO, YES-YES), Rp^2^ corresponds to the coefficient of PARTIAL determination, that is Rp^2^ is constructed based on the ratio of the variance explained by one risk factor over variance left unexplained by the controlling risk factors. Percentile 95% confidence intervals (CI) were obtained bootstraping, R = 2,000*,

(*) *indicating significant associations for which the bottom limit of the 95% CI must be positive. Rows in bold contain coefficients of determination for entire groups of predicting factors, indented rows (not bold) only for a single predicting factor*.

Some of our models include numerous covariates whose mutual linear dependency was expected to be strong, but it is precisely one of our main goals to assess the explanatory power of each of them as we hold the others constant (adding them as controlling variables). Further information on the collinearity between predictors can be found in the Supplementary Material in the form of a cross-correlation plot and an estimation of the Variance Inflation Factor (VIF) for each regression coefficient.

Because of a strongly skewed distribution of the error terms, we estimated 95% confidence intervals (CI) for our statistics directly re-sampling the observations and treating risk factors as random predictors (bootstrapping with R = 2,000 for each model). The exact R code necessary to replicate all the statistical analyses and figures has been made publicly available (http://dx.doi.org/10.17632/zy39mdzxpg.2).

## Results

Low self-rated general health and low MiL were found to be the most important factors associated with loneliness [R^2^ = 23.8%, CI (19.9%, 28.06%) and R^2^ = 24.87%, CI (21.62%, 28.45%), respectively]. Also, meaningful predictors of loneliness were low social connectedness and unhealthy lifestyles [R^2^ = 13.65%, CI (10.97%, 17%) and R^2^ = 8.09%, CI (5.88%, 11.09%), respectively]. Finally, socio-demographic factors presented very modest contributions [R^2^ = 0.6%, CI (0.15%, 1.72%)], barely reaching significance (see Figure 1A for a visual comparison and Table 2 for the corresponding R^2^ and Rp^2^ under different adjustments).

The predictive value of each factor was substantially reduced when we controlled for the other predictors. The total variance explained by general health became 5% [Rp^2^ = 7.37%, CI (5.07%, 10.43%)] instead of 23.9% (non-adjusted). Similarly, the contribution of MiL to loneliness became 4.8% [Rp^2^ = 7.04%, CI (4.87%, 9.67%)] instead of 25% (unadjusted). The contribution of social connectedness was reduced relatively less, only to one third, 5.9% [Rp^2^ = 8.54%, CI (6.39%, 11.4%)], indicating that its association with loneliness operates in a more independent manner. On the other hand, the contribution of lifestyles nearly disappeared, revealing that, under the supposition that there existed forward causal effects linking unhealthy lifestyles to loneliness, these effects would be totally mediated or confounded by the other risk factors. Further details on the particular contribution of each predictor within each conceptual group are provided below.

### Socio-Demographic Factors

Socio-demographic factors presented a strikingly low predictive power overall. We found that lower family incomes were associated with higher scores of loneliness, though the effect size was very small [R^2^ = 0.41, CI (0.02%, 1.2%)]. We did not find significant effects of sex, age, or education level on loneliness.

### Lifestyles

Except for nutrition, for which no significant association was found, all other lifestyles were able to predict loneliness in the anticipated direction: people with healthier habits (more exercise, better cognitive activity, and more restful and sufficient hours of sleep) were less likely to report high degrees of loneliness. Notably, the association with sleep quality was particularly strong [R^2^ = 6.68% CI (4.46%, 9.15%)], whilst exercise and cognitive activity, though significant, presented a much lower magnitude, not reaching 2% of the explained variance. It is worth noticing that these are full associations not controlling for any other risk factors. Upon adjusting for other risk factors, all these linear dependencies vanished to non-significance.

### Social Connectedness

This group made of two factors was able to explain around 14% of the total variance. Social interaction, measuring frequency and number of close social interactions, had a slightly higher explanatory power [R^2^ = 7.89%, CI (5.52%, 10.38%)] than household arrangement [R^2^ = 5.74%, CI (3.89%, 7.98%)]. Interestingly, the contributions of social interaction and household arrangement were largely independent from one another (see Figure 1). Regarding household arrangement, we found higher values of loneliness in participants living alone [mean UCLA score = 4.28, CI (4.14, 4.43)] or living in mono-parental households [mean = 4.06, CI (3.9, 4.24)], in comparison to individuals living with a partner with or without children in charge [mean = 3.57, CI (3.52, 3.63)].

### Self-Rated General Health

Both with and without adjusting for other risk factor groups, self-rated general health occupies a comparatively predominant role. The remarkable difference between adjusted and non-adjusted contribution is mainly due to the presence of predictors from the MiL group and, to a lesser extent, to the social connectedness group (see Venn diagram from Figure 2, left). Clearly, mental health is the main contributor within self-rated general health [R^2^ = 21.78%, CI (18%, 25.87%)], well above cognitive and physical health [R^2^ = 12.34%, CI (9.29%, 15.76%) and R^2^ = 7.79%, CI (5.62%, 10.28%) respectively]. Furthermore, when we partialed out the three predictors by each other to test for independent contributions it was evident that only mental health retains a strong association [Rp^2^ = 10.55%, CI (7.59%, 14.2%)], in sharp contrast with the very modest non-overlapping contributions of physical and cognitive health (see Table 2).

### Meaning in Life

The explanatory contribution of MiL is comparable in effect size to that of general health. Likewise, the association of MiL with loneliness strongly shrank after adjusting for the other risk factor groups, owing to a large overlap with general health and social connectedness (see Figure 2, left). As to the particular contribution of the three components in the MiL group, sense of coherence (SoC) clearly stood out above the other two, both before and after controlling for the other risk factor groups (see Figure 1 and related statistics in Table 1). Moreover, SoC almost completely accounted for all the contribution of the other two components, the opposite not being true. In other words, SoC, the most “cognitive” component of MiL, would still be considerably associated with loneliness even if participants did not present differences in purpose in life or engagement with life (see the overlapping pattern in the Venn diagram of Figure 2, right).

## Discussion

In this cross-sectional study, we observed a strong relationship between low scores of meaning in life (MiL) in adulthood and high feelings of loneliness. Its magnitude proved to be of the same order as the other well-known risk factors for loneliness, namely general health and social connectedness. By contrast, socio-demographic factors and lifestyles, with the exception of sleep quality, were very little predictive of loneliness.

There are numerous observational studies and surveys establishing general health status, especially mental health and poor social connectedness as the two key risk factors for loneliness. However, studies investigating potential psychological determinants tend to be scarcer and are seldom conducted in comparison with these and other major public health and socio-demographic predictive factors. The strong relationship we found between loneliness and MiL, including the motivational and cognitive underpins of this construct, suggests that efforts to combat loneliness should include interventions aiming at the consolidation of a structured meaningful vital plan.

In our study, additional models adjusting for predictors from within the same conceptual group (e.g., association with physical health holding mental health constant, etc.) were also informative. For general health, we found that mental health alone accounted for nearly all the dependency of loneliness with physical and cognitive health. By contrast, in the social connectedness group, household arrangement (those with whom we live) and social interactions (number and frequency with family and friends) hold independent associations with loneliness. An interesting finding is that adults living with children without a partner (mono-parental) are at a higher risk of feeling lonely than those with a partner, in the same degree as those living alone. This suggests that the key determinant of loneliness regarding household arrangement might not be living alone but living without a partner.

Regarding the MiL group, even though the three subcomponents investigated were strongly linked to loneliness, sense of coherence (SoC) was found to account for all the variance related to the others: purpose in life and engagement with life. This is a remarkable finding. SoC is the most cognitive dimension of MiL, introduced by the health sociologist Aaron Antonovsky to explain why certain individuals seemed to be particularly resilient, capable of maintaining themselves in a state of health, despite enduring adverse situations, such as poverty, marginality, or immigration (Antonovsky, 1990). SoC was constructed to measure an individual's belief that (1) inner and outer stimuli are structured, predictable, and explicable; (2) resources are available to meet the demands posed by these stimuli; and (3) these demands are challenges, worthy of investment. Hence, SoC is believed to strongly depend on the existence of values or principles that order and guide an individual's behavior and efforts.

Previous observational studies relating loneliness to psychological assessments similar to our MiL questionnaires have generally collected and analyzed data favoring a causal direction where loneliness is assumed to reduce a person's meaning in life. For instance, studies have asked people for their “sources” of meaning in life and found personal relationships and family as particularly crucial (Debats, 1999; Lambert et al., 2010). Indeed, manipulative psychological experiments have demonstrated that conditioning feelings of social belonging can modulate our capacity to find worth and meaning in life (Stillman et al., 2009; Lambert et al., 2013). However, nothing precludes the opposite causal direction from existing, that is, meaning in life may protect us from loneliness (Stillman and Lambert, 2013). In that sense, Rokach (2001) showed that one of the most important self-rated strategies for coping with loneliness (perceived causality) is “reflection and acceptance,” this strategy being particularly helpful among older people (Rokach, 2001). After all, researchers have often highlighted that it is not the lack of social connections that leads to loneliness, but the lack of meaning in them. It could then well be that the lack of meaning in our social bonds may arise or be aggravated by a lack of meaning in other spheres of life, being difficult to efficiently intervene one ignoring the others.

We found very large overlaps in the variance of loneliness explained by health status, social connectedness, and MiL. These intricated statistical dependencies are indicative of complex causal pathways (presence of confounding and mediated effects), that our observational data do not allow us to distinguish. Nor can we establish a forward causality direction linking low MiL to high loneliness. Consequently, and in the absence of an established theory mechanistically linking them, we have refrained from formulating any path analyses or structural equation model that would have favored one out of so many potential causal pathways. Nevertheless, the striking strength of our correlational results, even after adjusting for the other predictors, strongly invite testing cognitive interventions to combat loneliness that encompass reflection, reasoning, identification of personal values, and the comprehension of individuals' social and vital situation (e.g., acceptance and commitment therapy, mindfulness, etc.). If such programs were to succeed, they would have major implications for the future management and prevention of loneliness as a global condition.

Indeed, current governmental and other institutional programs to combat loneliness mainly employ situational and behavioral initiatives to re-wire socially isolated individuals, train or re-train social skills, and address maladaptive social cognition (Young, 1982; McWhirter, 1990; Perese and Wolf, 2005). However, previous studies and scientific meetings have already stressed the limitations of focusing exclusively on promoting social contact and connectedness (Masi et al., 2011) and identifying risk groups based on health status and socio-economic factors alone, not tailoring interventions to specific root causes (Fried et al., 2020). Our study provides observational evidence for the importance of incorporating actions to challenge how a lonely person understands his or her own life and obtains meaningful experiences from what he or she already possesses.

From the viewpoint of aging studies, although we found no differences in the prevalence of loneliness across the limited age segment of our cohort (40–68) in line with numerous surveys reporting a flat plateau in adulthood only limited at both ends by higher prevalence in adolescence and advanced age (Yang and Victor, 2011), public health studies and initiatives working on the impact of loneliness in elderly people should take notice of our results. During advanced age, key transitions linked with loneliness start accumulating, such as retirement from work, children leaving home, chronic health problems, bereavement, or entry into long-term care. To adopt the classification used in our study, we can identify most of these risk factors as belonging to changes of either social connectedness or personal health. By contrast, no negative changes from the meaning in life group seem to take place in old age. Indeed, Antonovsky believed SoC to remain stable after age 30 (Antonovsky, 1987) and more recent studies have even found that SoC improves over time (Feldt et al., 2011). With this and the above results in mind, it is suitable to speculate that strategies to improve and maintain MiL offer a strategically key target among the elderly in particular.

Our study has a number of limitations. First, the study cohort is not entirely representative of the Catalan population. We have an overrepresentation of women (67 vs. 51% in the Catalan population), of higher educated (73 vs. 35%), and of higher income participants (a 24% higher average net income). Nonetheless, within our sample, loneliness scores showed little statistical dependency with these socio-demographic factors and our main results were not affected when controlling for them, facilitating their generalizability to other populations.

Second, our models assumed linear functional relationships between our loneliness scores and different quantitative variables as we wished to capture first order main effects, whilst finer details could have been captured with higher order expansions and interaction terms affording more flexibility but at a higher risk of overfit. Third, our observational analysis only contains a snapshot of loneliness levels at a given point during a person's adulthood. Therefore, we were not able to distinguish between chronic or transient loneliness, to which MiL may be differently associated.

Finally, we did not evaluate the role of personality traits or specific medical conditions in modulating the relation between MiL components and loneliness. Further studies may take these variables into account and explore their potential role as little or non- modifiable confounders of the strong relationship we found between MiL and loneliness. If so, this would indeed limit the extent to which interventions promoting MiL might impact favorably on reducing loneliness.

In conclusion, our study emphasizes the relevance of considering aspects of individuals' psychological structure as complementary determinants of loneliness, in addition to well-known risk factors such as social connectedness and mental health. We also quantified the considerable overlap between these cognitive psychological constructs and mental health as predictors of loneliness. Further interventional studies and pilot programs are required to establish a forward causality between improvements in Meaning in Life, especially cognitive scaffolds ensuring comprehension (largely measured by SoC), and decreases in loneliness. If successful, we will gain in the definition and promotion of an individual's meaningful coherent vital plan a powerful new weapon in the battle our modern societies are waging against loneliness.

## Data Availability Statement

The datasets presented in this study can be found in online repositories. The names of the repository/repositories and accession number(s) can be found below: https://data.mendeley.com/datasets/zy39mdzxpg/2.

## Ethics Statement

The studies involving human participants were reviewed and approved by Comité d'Ètica i Investigació Clínica de la Unió Catalana d'Hospitals. The patients/participants provided their written informed consent to participate in this study.

## Author Contributions

AP-L, JT, and DB-F: securing funding sources. DM, GC, and JS: data collection. DM and GC: data analysis. DM and GC: had full data access to all data in the study and took responsibility for the integrity of the data and the analyses' accuracy. DM and DB-F: drafting of the manuscript. DM, DB-F, AP-L, and GC: review and writing contribution. All authors: concept, design, critical revision, and supervision.

## Conflict of Interest

The authors declare that the research was conducted in the absence of any commercial or financial relationships that could be construed as a potential conflict of interest.
