# Supplementary material for: Meaning in Life: A Major Predictive Factor for Loneliness Comparable to Health Status and Social Connectedness
Source: Front Psychol. 2021 Feb 24;12:627547. doi: 10.3389/fpsyg.2021.627547 (PMC7943478; doi:10.3389/fpsyg.2021.627547)
Supplement: Supplementary file 1 [file Table_1.DOCX]

# Supplementary Material

The present study involves models with numerous covariates whose linear dependency is well known and indeed expected. The rationale for building separate models with different number of covariates as we did in the main manuscript precisely lies in our interest to assess the explanatory power of each of them as we hold the others constant (that is, by adding them as controlling variables). However, a further clarification on the linear dependency of all the predictors with one another is illustrated below with their cross-correlation matrix. The impact of such multi-collinearity on the uncertainty of each regression coefficient estimation can be seen in its Variance Inflation factor (VIF) that we reported on the side. As we can see, no VIF reaches unacceptable values.


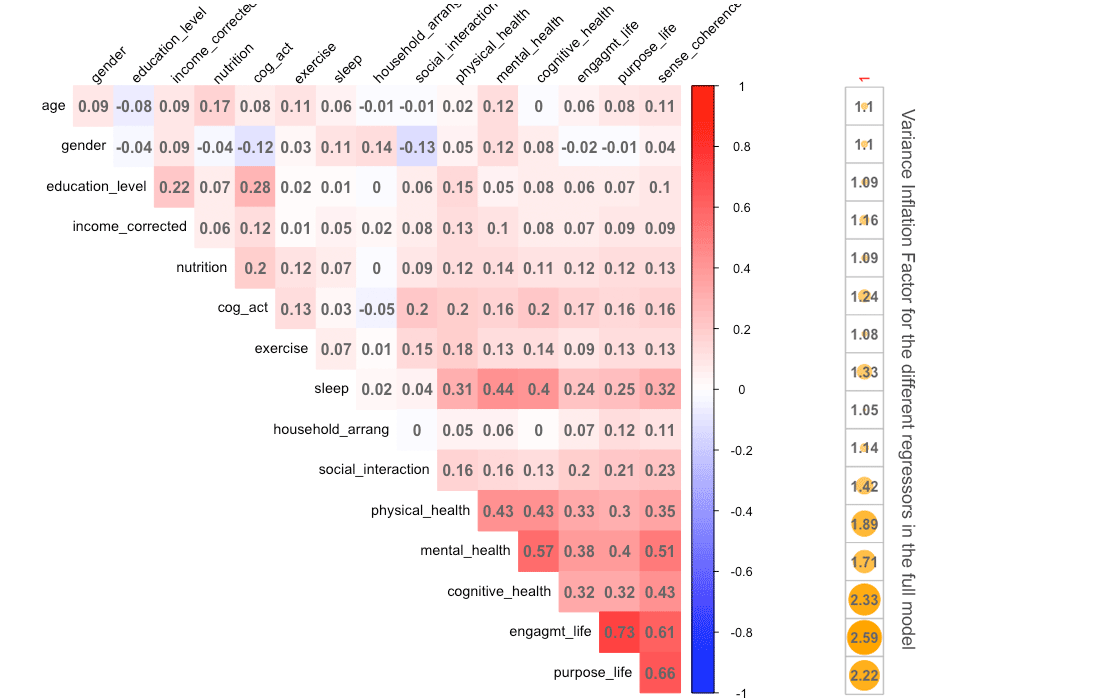


**Figure 1.** Cross-correlations on the left require numerical variables so that categorical factors were transformed to binary variables to enter the plot. As for gender, male was coded as 1 and female 0; exercise: low IPAQ corresponds to 0 whilst MEDIUM and HIGH to 1; and Household Arrangement was binarized so that people living with no partner (with children in charge or not) correspond to 0, otherwise to 1. When more than two levels were present in one factor, we chose the binarization of the levels so as to maximize the variance explained by the factor.
